# Loss of *Tpl2* activates compensatory signaling and resistance to EGFR/MET dual inhibition in v-RAS transduced keratinocytes

**DOI:** 10.1371/journal.pone.0266017

**Published:** 2022-03-24

**Authors:** Mary B. Kelley, Taylor J. Geddes, Maria Ochiai, Noah M. Lampl, W. Wade Kothmann, Sara R. Fierstein, Victoria Kent, Kathleen DeCicco-Skinner

**Affiliations:** Department of Biology, American University, Washington, DC, United States of America; Marshall University, UNITED STATES

## Abstract

Cutaneous squamous cell carcinoma (cSCC) is the second most common form of skin cancer in the United States, affecting one million people per year. Patients with aggressive disease have limited treatment options and high mortality, highlighting the need to identify new biomarkers linked to poor clinical outcome. HRAS mutations are found in skin papillomas and cSCCs and increase in frequency when MAP3K family members are inhibited, suggesting a link between blockade of mitogen-activated protein kinase (MAPK) signaling and initiation of RAS-primed cells. *Tpl2*, a MAP3K gene, can serve as a tumor suppressor gene in cSCC. We have previously shown that upon *Tpl2* ablation, mice have heightened sensitivity to aberrant RAS signaling. *Tpl2*^-/-^ mice display significantly higher numbers of papillomas and cSCCs in two-stage chemical carcinogenesis studies and increased tumorigenicity of keratinocytes expressing oncogenic v-ras^Ha^ in nude mouse skin grafts. In part, this is mediated through increased mesenchymal-epithelial transition factor (MET) receptor activity. Epidermal Growth Factor Receptor (EGFR) is reported to be an essential factor for MET-driven carcinogenesis and MET activation may confer resistance to EGFR therapies, suggesting that the concurrent use of both an EGFR inhibitor and a MET inhibitor may show promise in advanced cSCCs. In this study we assessed whether normal or Ras-transformed *Tpl2*^-/-^ keratinocytes have aberrant EGFR signaling and whether concomitant treatment with EGFR/MET tyrosine kinase inhibitors was more effective than single agents in reducing growth and angiogenic potential of Ras-transformed keratinocytes. *Tpl2*^-/-^ keratinocytes exhibited increased HER-2 and STAT-3 under basal conditions and elevated p-MET and p-EGFR when transduced with oncogenic RAS. Inhibition of MET by Capmatinib increased p-EGFR in *Tpl2*^*-/-*^ keratinocytes and papillomas, and inhibition of EGFR by Gefitinib increased HER2 and HER3 signaling in both genotypes. Treatment of keratinocytes with EGFR and MET inhibitors, in combination, significantly enhanced endothelial tube formation, MMP-9 activity and activation of other RTKs, with more pronounced effects when *Tpl2* was ablated. These data indicate that Tpl2 cross-talks with both EGFR and MET signaling pathways. Upon inhibition of EGFR/MET signaling, a myriad of escape mechanisms exists in keratinocytes to overcome targeted drug effects.

## Introduction

More people are diagnosed with skin cancer each year than all other cancers combined [1]. Cutaneous squamous cell carcinoma (cSCC), a form of non-melanoma skin cancer, accounts for 20% of all cutaneous malignancies, and its incidence is rising at an alarming rate [2,3]. Although primary cSCC cases are non-metastatic and can be well-managed with surgical treatment, patients with unresectable or metastatic cSCC have limited treatment options, resulting in an overall five-year survival rate of 26% [4].

Cutaneous SCCs arise from malignant proliferation of keratinocytes found in the epidermis of the skin [5]. UV- or chemically-induced mutations in keratinocytes can lead to genomic instability, allowing acquisition of additional mutations that can cause upregulation in growth and survival pathways [5]. Several laboratories have conducted whole exome sequencing of cSCCs in the hopes of identifying mutational signatures for aggressive disease [6,7]. While metastatic cSCCs harbor a very high mutational burden and a complicated genetic landscape, most cSCC mutations are found in genes involved in the RAS/MAPK, NF-κB, and PI3K/AKT pathways [7,8]. Along these lines, activating mutations in *HRAS* have been found in 3–20% of advanced cSCCs [6,7,9].

RAS proteins (K-RAS, N-RAS, H-RAS) are small GTPases that lie upstream of the mitogen-activated protein kinase (MAPK) signal transduction cascade [10]. Ligand binding, and activation of RAS, stimulates a three-tier cascade system whereby a MAP3K phosphorylates/activates a MAP2K, which phosphorylates/activates MAPK. MAPK enters the nucleus and activates a diverse array of genes involved in growth, survival, differentiation, and inflammation [11].

There are at least twenty members of the MAP3K family [12]. Elevated MAPK activity has been found in numerous tumor types, leading to the development of a variety of MAPK inhibitors for the treatment of cancer and inflammatory diseases [13]. Unfortunately, inhibition of MAP3K family members in melanoma patients has often led to paradoxical activation of compensatory feed-back loops, which drives drug resistance and results in secondary cSCC formation [14,15]. The cSCCs that develop in these patients have a substantially increased frequency of *HRAS* mutations, suggesting that inhibition of MAP3K signaling causes an increase in selection and expansion of RAS-mutated cells [16,17].

The role of *HRAS* in the initiation of skin carcinogenesis can be assessed using both *in vivo* and *in vitro* methodologies [18]. One of the best established *in vivo* models to study the development of cSCCs is the two-stage chemical carcinogenesis model in mice [19,20]. In this model, mice are initiated with 7,12-dimethylbenz(a)anthracene (DMBA) which creates an irreversible and specific mutation in codon 61 of *HRAS* [21]. This initiation is followed by twice weekly application of the phorbol ester 12-O-tetradecanoylphorbol-13-acetate (TPA). Typically, benign papillomas arise within 10–15 weeks, and a small percentage progress to cSCCs [19]. The preponderance of tumors contain mutations in the *HRAS* oncogene, and show remarkable phenotypic similarities with human cSCCs [19]. The ability of *HRAS* to initiate tumorigenesis in keratinocytes *in vitro* has also been established [22]. Keratinocytes infected with a v-ras^Ha^ oncogene using a replication-defective retrovirus have a high proliferation rate, don’t terminally differentiate in response to high calcium, and generate papillomas when grafted on nude mice [23].

Tumor progression locus 2 (*Tpl2*), a MAP3K family member, is a critical regulator of oncogenic and inflammatory pathways [24]. We have previously reported a tumor suppressor function of *Tpl2* in skin [25,26]. *Tpl2*^-/-^ mice develop significantly more skin papillomas and cSCCs compared to wild type counterparts, have a greatly reduced tumor latency and more progressive disease [25–28]. Further, we have shown that loss of *Tpl2* in mice, increases susceptibility to the oncogenic effects of RAS. *Tpl2*^-/-^ keratinocytes expressing oncogenic v-ras^Ha^ proliferate 44% faster than v-ras^Ha^-transduced wild type cells, are three times more invasive, have heightened matrix metalloproteinase levels, and undergo significantly more *in vitro* malignant conversion [25,27]. *In vivo*, tumorigenicity of keratinocytes expressing oncogenic v-ras^Ha^ in nude mouse skin grafts was nine fold higher when keratinocytes and fibroblasts were missing the Tpl2 gene [26].

In the absence of Tpl2, several RTK mediated bypass pathways become activated, contributing to skin tumorigenesis [24–26,29]. Among these pathways is the hepatocyte growth factor (HGF)/mesenchymal-epithelial transition factor (MET) signaling cascade, a pro-survival pathway linked to tumor growth, invasion and metastasis [30]. Overexpression of MET is found in *Tpl2*^-/-^ keratinocytes and cSCCs, and pharmacological inhibition of MET in *Tpl2*^-/-^ mice decreased overall tumor burden by 60% and prevented malignant conversion of papillomas to cSCC [25]. However, MET monotherapy in *Tpl2*^-/-^ mice does not completely abolish tumor growth, suggesting that tumor survival mechanisms that bypass MET inhibition may help maintain oncogenic signaling.

Both RAS and MET can initiate squamous cell carcinogenesis through activation of epidermal growth factor receptor (EGFR) [31]. Further, EGFR is reported to be a critical player in MET-driven carcinogenesis, as blocking EGFR causes MET-driven tumors to regress [31]. The EGFR family has four members including ErbB1 (EGFR or HER1), ErbB2 (HER2), ErbB3 (HER3), and ErbB4 (HER4) [32]. EGFR can heterodimerize with its family members HER2 and HER3 [33]. These receptors are often co-expressed in cSCCs and are implicated in skin cancer progression and poor clinical outcome [31,34–37].

The current study tested whether concomitant inhibition of both EGFR and MET was more effective than single use agents in reducing keratinocyte growth, *in vitro* angiogenesis, downstream signaling molecules, and matrix metalloproteinase-9 (MMP-9) activity in normal and Ras-transformed keratinocytes in wild type and Tpl2 null mice. In the presence of oncogenic Ras, EGFR and MET combination treatment increased MMP-9 activity and endothelial tube formation, with more pronounced effects when Tpl2 was ablated. Further, Ras-transformed keratinocytes treated with EGFR and MET inhibitors, in combination, activated an intricate network of receptor detours which could help maintain survival/progression signals in cSCC.

## Results

### EGFR/HER2/HER3 family members are dysregulated in *Tpl2*^-/-^ keratinocytes and increased in a compensatory fashion upon treatment with Gefitinib

**Fig 1:**
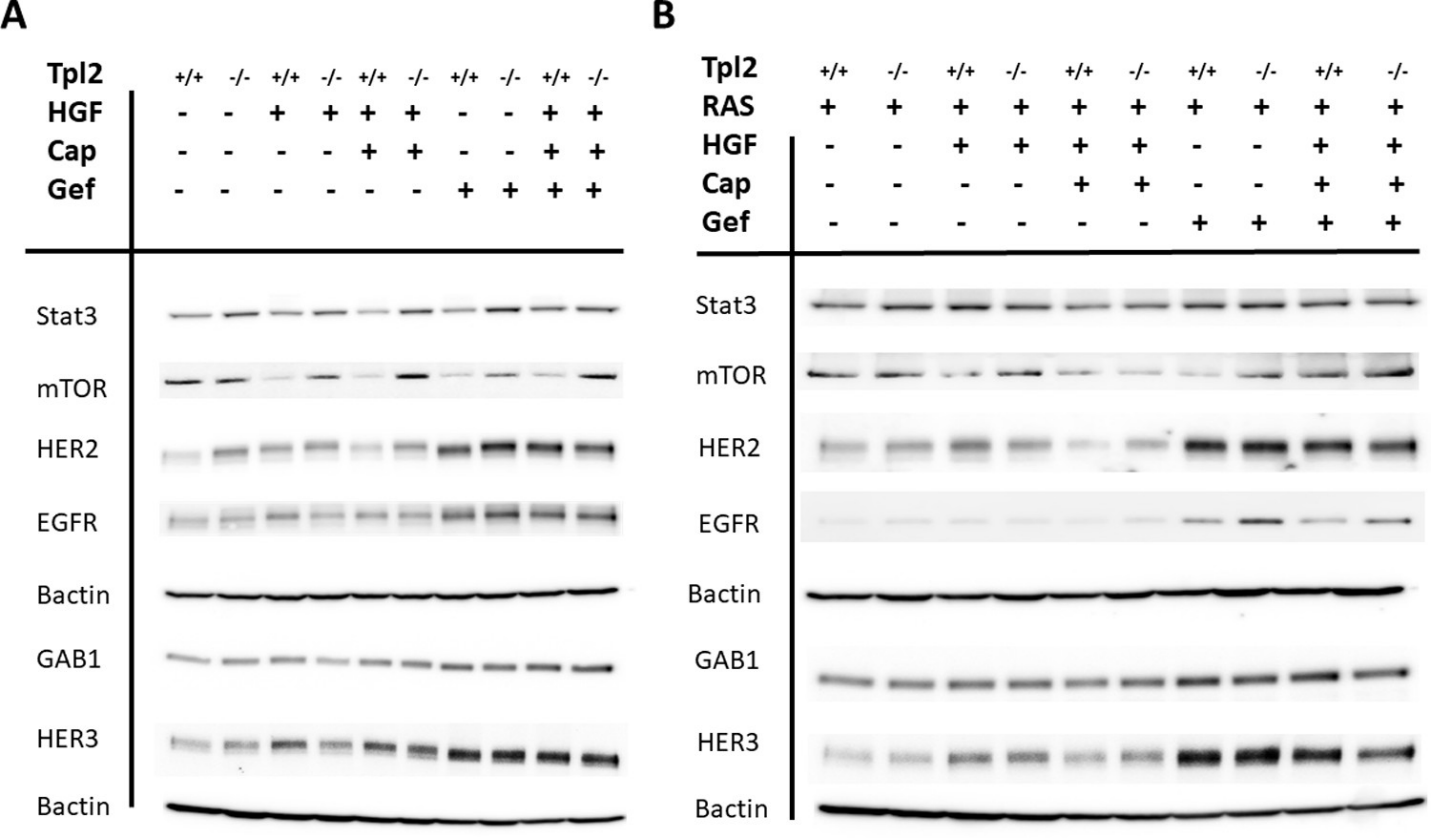
Western analysis of normal and *v-ras*^*Ha*^-transduced wild type and *Tpl2*^-/-^ keratinocytes. Cells were cultured alone (A) or initiated with *v-ras*^*Ha*^-retrovirus (B) for 36 hours. Wild type or *Tpl2*^-/-^ keratinocytes were then treated with 20ng/ml HGF, 2nM Capmatinib + HGF, 1uM Gefitinib, or Gefitinib/Capmatinib/HGF and lysates collected 24 hours later. All Westerns were performed a minimum of three times.

We have previously reported that *Tpl2*^-/-^ mice have increased HGF/MET signaling in isolated keratinocytes, skin and cSCCs, which contribute to skin tumorigenesis and progression [25]. MET is reported to cooperate with EGFR to induce mouse and human cSCCs [31]. To assess the crosstalk between EGFR and MET, and how this relates to *Tpl2* status, we performed Western analyses. In *Tpl2*^-/-^ keratinocytes, HER2 protein levels were 2.5-fold higher under basal conditions than wild type keratinocytes (Figs 1A and S2). HGF-stimulation increased HER2 protein 1.8-fold in wild type cells, which could be partially blocked (49%) with Capmatinib, a pMET inhibitor. In an opposing fashion, HER2 levels in *Tpl2*^-/-^keratinocytes were resistant to both HGF-stimulation and inhibition by Capmatinib. Gefitinib, which blocks EGFR phosphorylation, induced compensatory HER2 signaling in both wild type and *Tpl2*^-/-^ keratinocytes, yielding increases of 3.1 and 3.9-fold respectively. STAT-3 also showed genotype differences between wild type and *Tpl2*^-/-^ keratinocytes, with *Tpl2*^-/-^ keratinocytes having 1.5-fold higher protein levels under basal conditions than wild type cells (Fig 1A). However, STAT-3 protein levels weren’t altered upon treatment with HGF, Capmatinib or Gefitinib in either genotype. Untreated keratinocytes from wild type or *Tpl2*^-/-^ mice had similar mTOR protein levels. However, treatment with Capmatinib or the combination of Capmatinib/Gefitinib decreased mTOR 90% in wild type keratinocytes. mTOR protein decreased 55% in Gefitinib-treated *Tpl2*^-/-^ keratinocytes, but dual treatment with Capmatinib/Gefitinib restored mTOR expression to levels that were similar to baseline values. Total EGFR, HER3, and Gab1 proteins showed similar expression patterns. In all cases, HGF-stimulation increased total protein (1.8, 2.3, 1.3-fold respectively) in wild type cells but not in *Tpl2*^-/-^ keratinocytes. Gefitinib treatment increased EGFR, HER3, and Gab1 total protein (3, 3.8, 2.5-fold respectively) in wild type cells and increased total protein (2.9, 4.6, 2.7- fold respectively) in *Tpl2*^-/-^ keratinocytes. Concomitant treatment of keratinocytes with Capmatinib + Gefitinib stimulated EGFR, HER3, and Gab1 protein levels (2.7, 5.1, 3.3-fold in wild type keratinocytes vs. 3.5, 5.1, 4-fold in *Tpl2*^-/-^ keratinocytes respectively).

RAS mutations are associated with the development of cSCC in both mice and humans [18,38]. For some experiments, keratinocytes were infected with a replication-defective *v-ras*^*Ha*^ retrovirus prior to treatment. This initiates them in a manner similar to both human cSCC and mouse DMBA/TPA two-stage carcinogenesis studies. *v-ras*^*Ha*^-transduction prior to treatment with HGF, Gefitinib and/or Capmatinib eliminated the basal genotypic differences in HER2, HER3, and STAT-3. Similar to what we found in non-transduced keratinocytes, treatment of wild type *v-ras*^*Ha*^-transduced keratinocytes with Gefitinib increased EGFR, HER2, HER3 and Gab1 4.9-fold, 2.5-fold, 9-fold, and 2.2-fold respectively (Fig 1B). Treatment of *Tpl2*^-/-^ *v-ras*^*Ha*^-infected keratinocytes with Gefitinib increased EGFR, HER2, HER3 and Gab1 10-fold, 2.4-fold, 9.1-fold and 2.2-fold respectively. Treatment with Capmatinib decreased the HGF-stimulated induction in HER2 (5.4-fold vs. 1.5-fold), HER3 (1.7-fold vs no change), STAT-3 (1.7-fold vs. 1.3-fold), and mTOR (no change vs 3.8-fold) in wild type vs. *Tpl2*^-/-^ keratinocytes respectively. There were no additive effects when Capmatinib + Gefitinib were administered together compared to Gefitinib alone.

### Inhibition of MET phosphorylation enhances p-EGFR and p-AKT signaling in *Tpl2*^-/-^ keratinocytes and p-EGFR in papillomas

**Fig 2:**
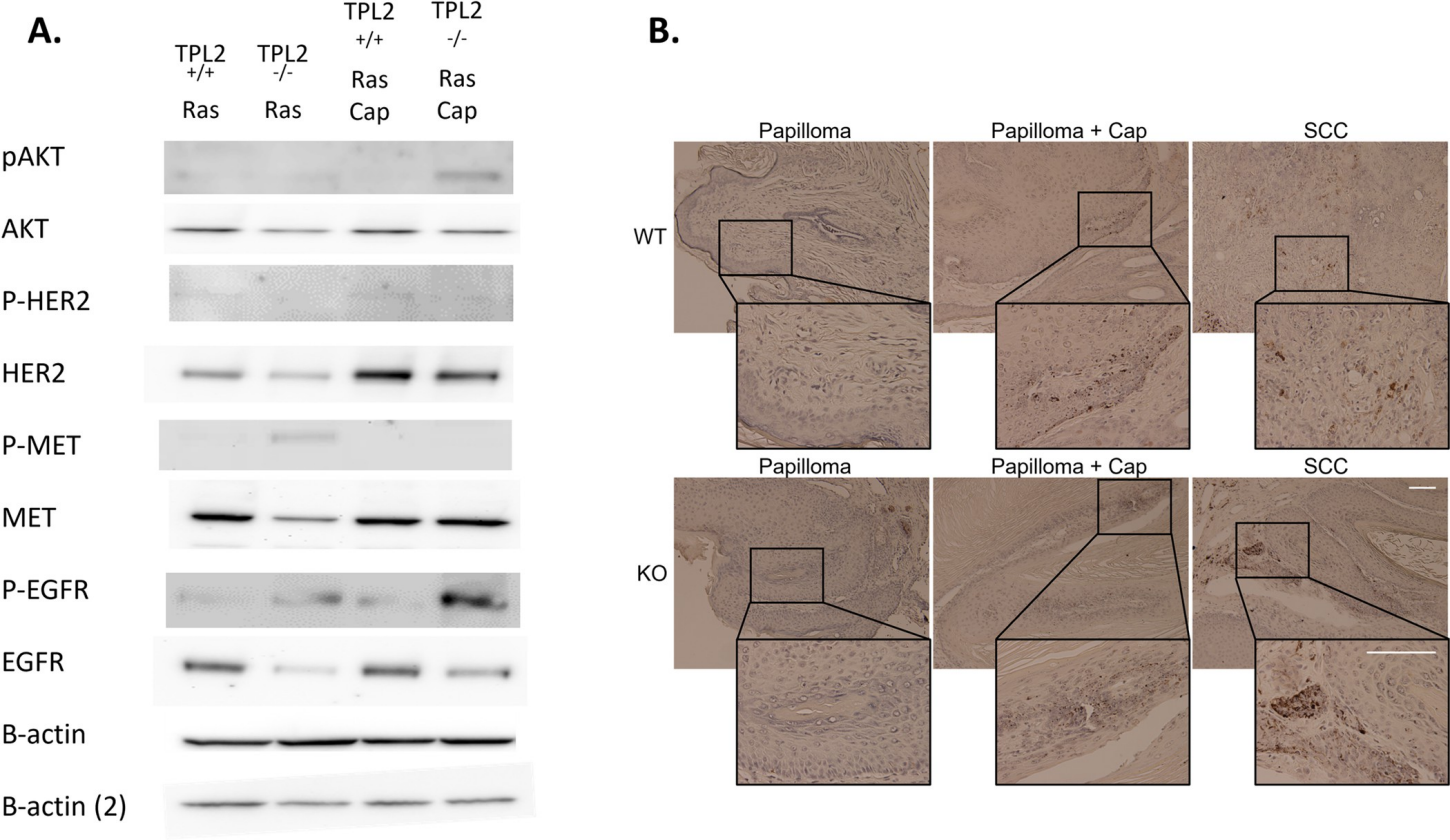
Inhibition of MET by Capmatinib enhances p-EGFR and p-AKT signaling in keratinocytes and p-EGFR in papillomas. (A) Cells were v-ras^Ha^ infected for 36 hours to express viral protein. Some keratinocytes were treated with 2 nM Capmatinib. Total protein was lysed 15 min post Capmatinib treatment and immunoblotted for p-AKT/AKT, p-HER2/HER2, p-MET/MET, p-EGFR/EGFR, and B-actin. (B) Papillomas and SCCs from wild type or *Tpl2* ^-/-^ mice, and papillomas from wild type or *Tpl2* ^-/-^ mice fed Capmatinib in their diet were stained for p-EGFR. Images at 10X and 50X are displayed.

*v-ras*^*Ha*^-transduced *Tpl2*^-/-^ keratinocytes have 5-fold higher p-MET and 2.8-fold higher p-EGFR compared to *v-ras*^*Ha*^-transduced wild type keratinocytes (Fig 2A). Blocking MET phosphorylation through Capmatinib increased p-EGFR expression 10-fold in *v-ras*^*Ha*^-transduced *Tpl2*^-/-^ keratinocytes but only 1.3-fold in *v-ras*^*Ha*^-transduced wild type keratinocytes. Capmatinib treatment also induced p-AKT 4.3-fold in *v-ras*^*Ha*^-transduced *Tpl2*^-/-^ keratinocytes. Additionally, p-EGFR was strongly induced in papillomas from Capmatinib-fed mice compared to papillomas from wild type and *Tpl2*^-/-^ mice on normal diet, and increased in SCCs compared to papillomas (Fig 2B). p-EGFR expression in SCCs from Capmatinib-fed mice could not be assessed, as no papillomas in this group underwent malignant conversion to become SCCs.

### Effect of Gefitinib and Capmatinib on the proliferation of v-ras^Ha^-transduced wild type and *Tpl2*^-/-^ keratinocytes

**Fig 3:**
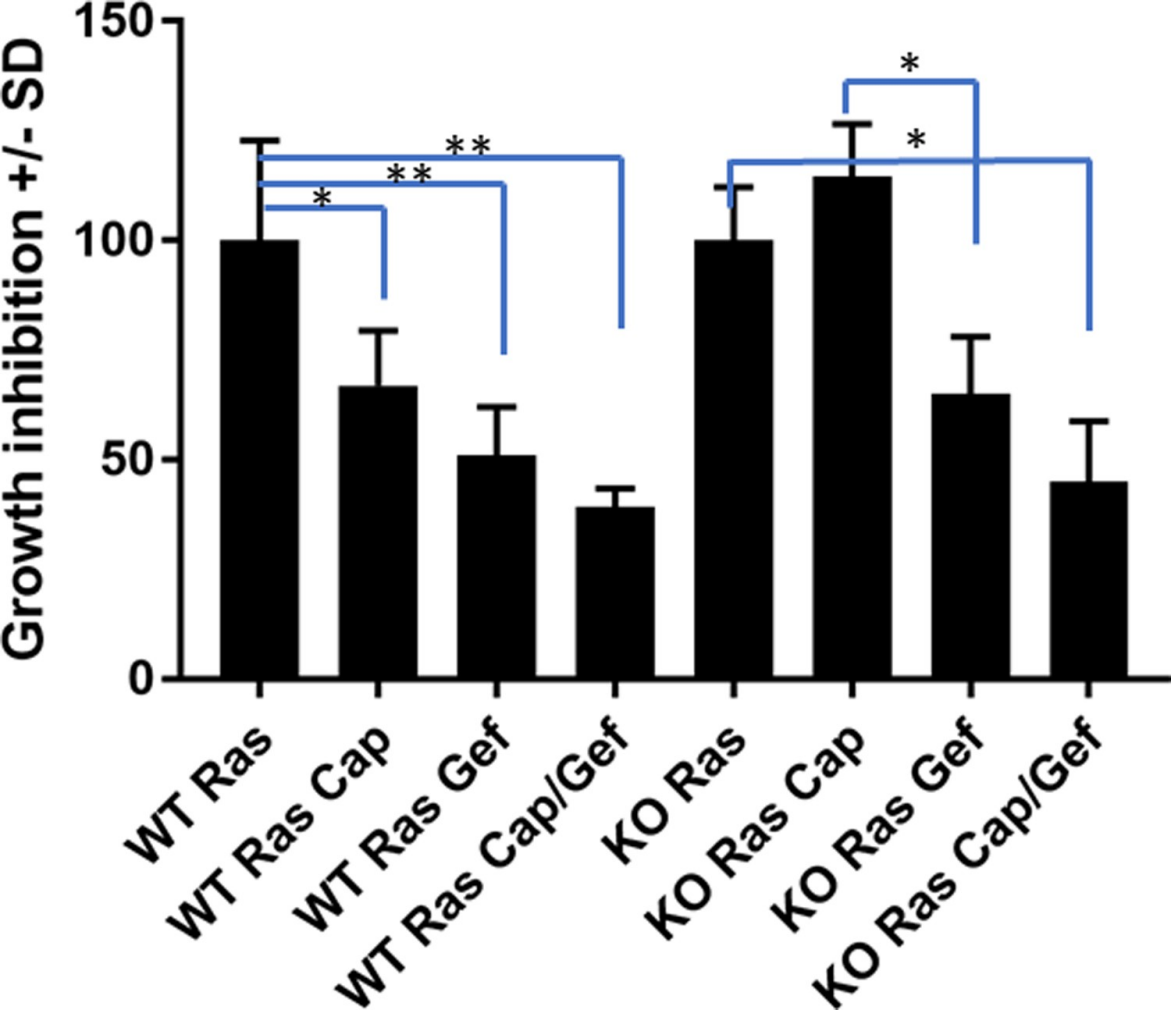
Gefitinib + Capmatinib decrease proliferation of *v-ras*^*Ha*^-transduced wild type and *Tpl2*^-/-^ keratinocytes. v-*ras*^Ha^-transduced Wild type or *Tpl2*^-/-^ keratinocytes were grown in the presence of 1uM Gefitinib, 2nM Capmatinib, or both Gefitinib and Capmatinib. Results of quadruplicate samples were normalized to respective controls (untreated v-*ras*^Ha^-transduced *Tpl2*^+/+^ (WT) keratinocyte cells for all WT samples or untreated v-*ras*^Ha^-transduced *Tpl2* ^-/-^ keratinocyte controls for KO samples) * p<0.05, **p<0.01.

Cultured mouse keratinocytes from wild type or *Tpl2*^*-/-*^ mice were initiated *in vitro* by the introduction of a replication-defective v-ras^Ha^ retrovirus and treated with Gefitinib +/- Capmatinib. Proliferation of *v-ras*^*Ha*^-transduced wild type cells was inhibited 33% with Capmatinib, whereas *Tpl2*^*-/-*^ keratinocytes were resistant to the anti-proliferative effects of Capmatinib (Fig 3). Gefitinib inhibited *v-ras*^*Ha*^-transduced wild type keratinocytes 48% and *Tpl2*^*-/-*^ keratinocytes 35%. Keratinocytes treated with both Capmatinib and Gefitinib had a 61% growth inhibition for wild type cells and 52% for *Tpl2*^*-/-*^ cells, although statistically this was no different than treatment with Gefitinib alone.

### Capmatinib/Gefitinib treatment increases MMP-9 activity, with more pronounced effects in Tpl2^-/-^ keratinocytes

**Fig 4:**
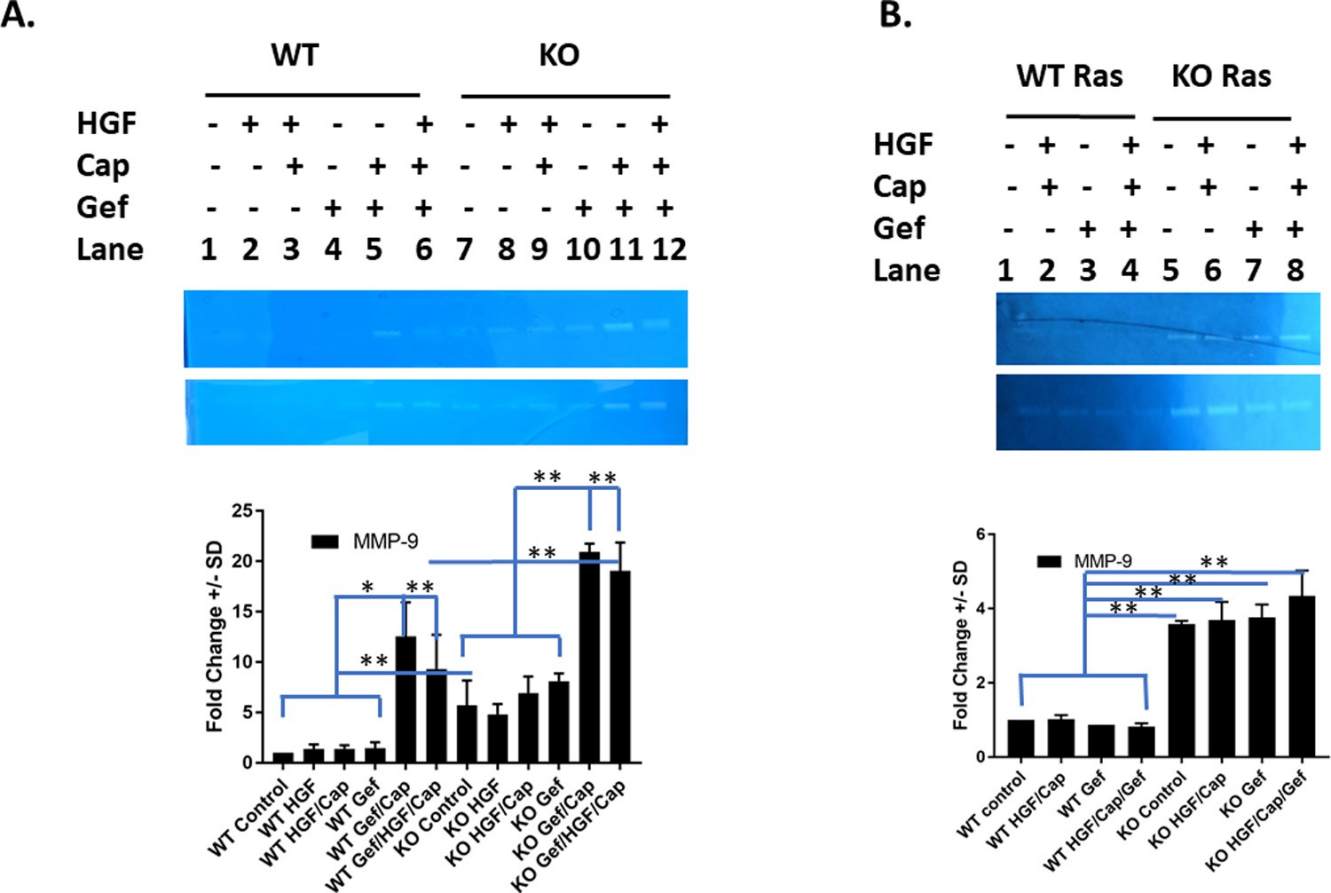
Capmatinib/Gefitinib treatment increases MMP-9 activity, with more pronounced effects in *Tpl2*^-/-^ keratinocytes. (A) Enzymatic activity of MMP-9 in conditioned media from wild type or *Tpl2*^-/-^ normal keratinocytes treated with 20ng/mL HGF, 20ng/ml HGF+ 2nM Capmatinib, 1uM Gefitinib, or all treatments in conjunction. Biological replicates from two different cohorts of wild type or *Tpl2*^-/-^ mice (n = 4/condition) are represented by two gels. (B) Enzymatic activity of MMP-9 in conditioned media from *v-ras*^*Ha*^-transduced wild type and *Tpl2*^-/-^ keratinocytes treated with 20ng/ml HGF+ 2nM Capmatinib, 1uM Gefitinib, and treatments in conjunction. Biological replicates from two different cohorts of wild type or *Tpl2*^-/-^ mice (n = 6/condition) are represented by two gels.

Increased MMP-9 enzymatic activity reflects invasiveness of a tumor, increased ability to metastasize and increased angiogenesis. To measure the proteolytic activity of MMP-9, zymography was performed using conditioned media from untreated and treated control and *v-ras*^*Ha*^-transduced keratinocytes. Under basal conditions, MMP-9 activity was barely detectable in wild type cells, and HGF/Capmatinib or Gefitinib treatment alone had little effect on MMP-9 activity (Fig 4A). However, the combination of Gefitinib/Capmatinib or HGF/Gefitinib/Capmatinib induced MMP-9 activity in wild type cells 12-fold and 9-fold respectively. MMP-9 activity was 5.7-fold higher in conditioned media from untreated *Tpl2* ^-/-^ keratinocytes compared to wild type cells (Fig 4A). The combination of Gefitinib/Capmatinib or HGF/Gefitinib/Capmatinib increased this MMP-9 activity even further, achieving levels 3.5-fold higher than untreated *Tpl2* ^-/-^ keratinocytes.

MMP-9 activity was also measured in *v-ras*^*Ha*^-transduced wild type and *Tpl2* ^-/-^ keratinocytes. MMP-9 activity was 3.6-fold higher in conditioned media from untreated *v-ras*^*Ha*^-transduced *Tpl2* ^-/-^ keratinocytes compared to *v-ras*^*Ha*^-transduced wild type cells (Fig 4B). These genotype differences were maintained, although *v-ras*^*Ha*^-transduction negated some of the treatment effects seen in normal keratinocytes.

### Capmatinib/Gefitinib treatment increases endothelial tube formation with more pronounced effects in *Tpl2*^-/-^ keratinocytes

**Fig 5:**
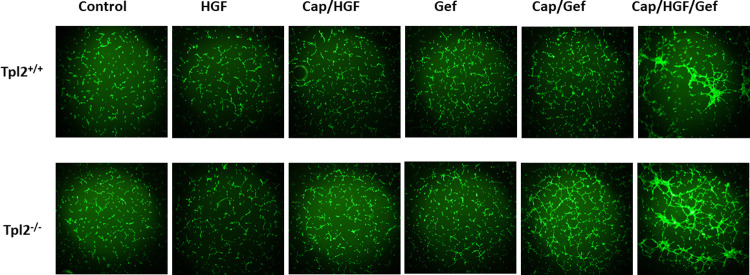
Keratinocytes treated with HGF/Gefitinib/Capmatinib develop more segments, nodes and mesh in an endothelial tube formation assay, with more pronounced effects in *Tpl2*^-/-^ cells. 3B11 mouse endothelial cells were serum starved overnight. 75,000 3B-11 cells/well were subjected to keratinocyte conditioned media from wild type or *Tpl2*^-/-^ cells treated with 20ng/ml HGF, HGF+ 2nM Capmatinib, 1uM Gefitinib, Capmatinib/Gefitinib, or HGF/Capmatinib/Gefitinib. Tube networks were grown for 8 hours and imaged under 4X magnification with a fluorescence microscope. The number of nodes, mesh, and segments were quantified using NIH Image J with Angiogenesis Plugin.

An endothelial tube formation assay is an *in vitro* method for determining factors involved in angiogenesis [39]. Serum starved and fluorescently labeled 3B-11 endothelial cells plated over basement membrane extract were subjected to conditioned media from untreated or treated control and *v-ras*^*Ha*^-transduced keratinocytes. Untreated wild type and *Tpl2* ^-/-^ keratinocytes typically produce few tubes due to a lack of secreted angiogenic factors, unless higher concentrations of 3B-11 cells are used (Figs 5 and S3). However, the tube network was substantially increased when conditioned media from Gefitinib/Capmatinib or HGF + Gefitinib/Capmatinib cells were used, with more pronounced effects in *Tpl2* ^-/-^ keratinocytes.

**Fig 6:**
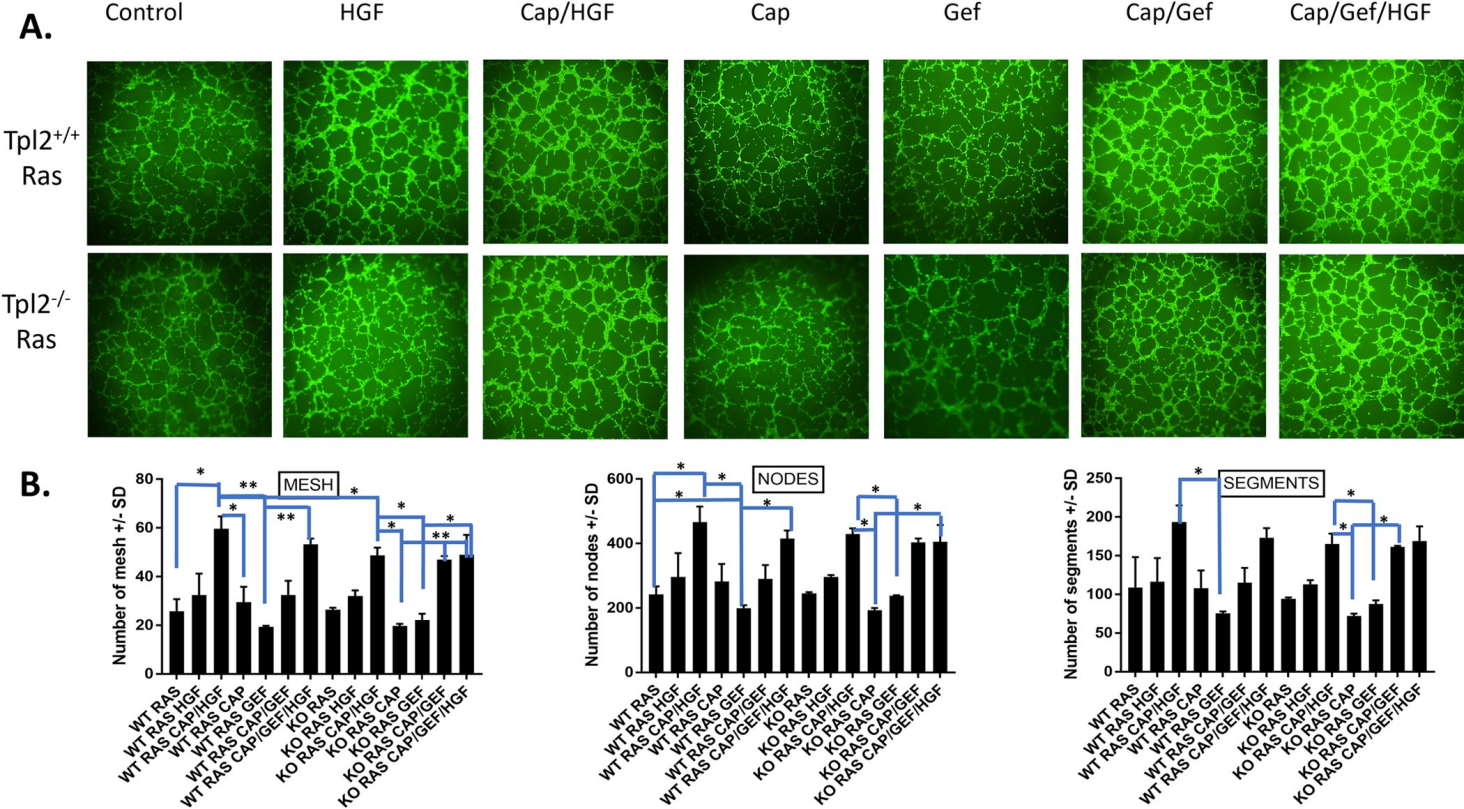
v-*ras*^Ha^-transduced wild type or *Tpl2*^-/-^ keratinocytes treated with HGF/Gefitinib/Capmatinib develop more segments, nodes and mesh in an endothelial tube formation assay. 3B11 mouse endothelial cells were serum starved and subjected to keratinocyte conditioned media from v-*ras*^Ha^-transduced wild type or *Tpl2*^-/-^ cells treated with 20ng/ml HGF, HGF+ 2nM Capmatinib, 1uM Gefitinib, Capmatinib/Gefitinib, or HGF/Capmatinib/Gefitinib. Tube networks were grown for 6 hours and imaged under 4X magnification with a fluorescence microscope. The number of nodes, mesh, and segments were quantified using NIH Image J with Angiogenesis Plugin.

Conditioned media from v-*ras*^Ha^-transduced wild type or *Tpl2*^-/-^ keratinocytes produce an extensive tube network, even under basal conditions (Fig 6A). The combination of Gefitinib/Capmatinib or HGF/Gefitinib/Capmatinib induced the number of segments, nodes and mesh 1.6–2.0-fold in both wild type and *Tpl2*^-/-^ keratinocytes (Fig 6B).

### v-*ras*^Ha^-transduced wild type and *Tpl2*^-/-^ keratinocytes treated with Capmatinib +/- Gefitinib have coactivation of multiple phospho-RTKs

**Fig 7:**
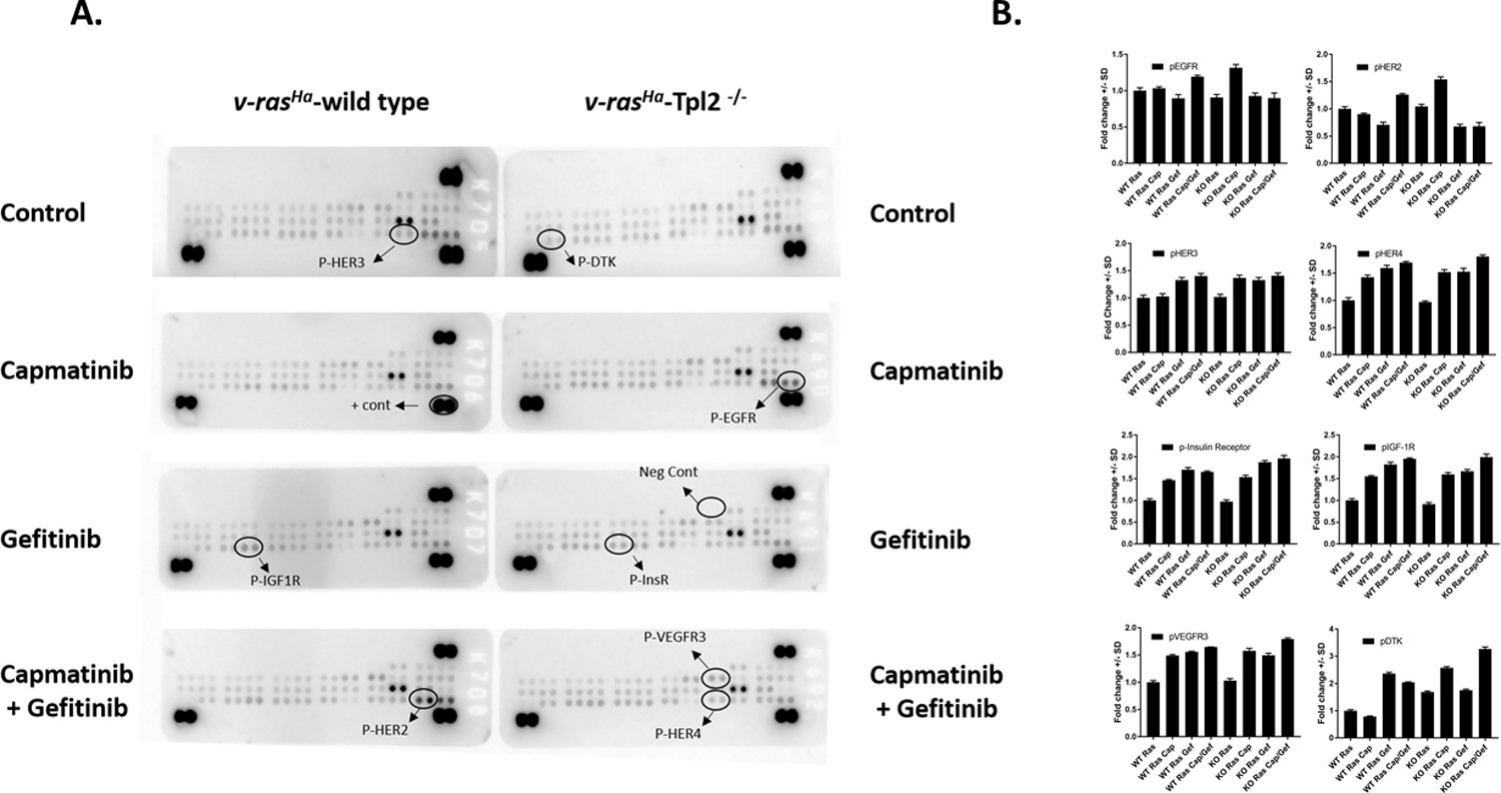
v-*ras*^Ha^-transduced *Tpl2*^+/+^ and *Tpl2*^-/-^ keratinocytes treated with Capmatinib +/- Gefitinib have coactivation of multiple phospho-RTKs. *v-ras*^*Ha*^-transduced wild type and *Tpl2*^-/-^ keratinocytes were treated with 2nM of Capmatinib, 1uM of Gefitinib, or 2nM Capmatinib +1uM Gefitinib for 15 minutes, after which point lysates were collected. 200ug/ml of cell lysates were applied to each array. Each RTK is spotted in duplicate, and the spots at each corner are positive controls. Blots (A) and graphs (B) of the most differentially expressed RTKs are displayed.

A phospho-RTK assay was performed in v-*ras*^Ha^-transduced wild type and *Tpl2*^-/-^ keratinocytes treated with Capmatinib +/- Gefitinib to determine expression across 39 phosphorylated RTKs. Capmatinib treated *Tpl2*^-/-^ keratinocytes had 1.4–2.3-fold increases in multiple other RTKs including p-EGFR, p-HER2, p-HER3, p-HER4, p-IGF-1, p-Insulin Receptor, p-VEGFR3 and p-DTK, whereas wild type cells treated with Capmatinib had 1.4–1.5-fold increases in p-HER4, p-IGF-1, p-Insulin Receptor, and p-VEGFR3 (Fig 7A and 7B). v-*ras*^Ha^-transduced wild type and *Tpl2*^-/-^ keratinocytes had 1.5–3.3-fold increases in p-HER3, p-HER4, p-IGF-1, p-Insulin Receptor, p-VEGFR3 and p-DTK when treated with Gefitinib or the combination of Capmatinib + Gefitinib (Fig 7A and 7B).

## Discussion

Treatment of metastatic cSCC remains a therapeutic challenge due to the high heterogeneity of the disease [8]. Members of the RTK/RAS/MAPK pathway are frequently mutated in cSCC [40]. Among them, HRAS can serve as an early event in cSCC development and its mutation frequency is increased upon inhibition of MAPK signaling [8].

*Tpl2* is a protein kinase in the MAPK cascade. As a MAP3K protein, *Tpl2* is downstream of RTKs, including EGFR, HER2/3 and MET, as well as the GTPase RAS. The loss of *Tpl2* activates MET signaling in skin, and increases MET-dependent skin tumorigenesis and progression [25]. Pharmacological inhibition of MET in *Tpl2*^-/-^ mice is somewhat effective, decreasing overall tumor burden and conversion to cSCC, but doesn’t completely eliminate tumor growth or restore numbers to the level of wild type controls [25]. This suggests that other survival mechanisms may be activated in the presence of MET inhibition. In other cancers, MET monotherapy has demonstrated limited effectiveness due to activation of bypass pathways such as EGFR and HER [41,42]. Thus, in this study, we assessed p-EGFR and HER2/3 in the presence of the MET inhibitor Capmatinib. We found upregulation in p-EGFR in v-*ras*^Ha^-transduced *Tpl2*^-/-^ keratinocytes and cSCCs compared to wild type cells. Inhibition of p-MET by Capmatinib further increased expression of p-EGFR in v-*ras*^Ha^-transduced *Tpl2*^-/-^ keratinocytes and papillomas, supporting the notion of bypass pathway activation.

The MET receptor can heterodimerize with EGFR, or its family members HER2 and HER3, to activate downstream PI3K/AKT, MAPK or JAK/STAT signaling [33,43]. We found higher expression of HER 2 and lower expression of HER 3 in *Tpl2*^-/-^ keratinocytes under basal conditions. However, both HER2 and HER3 receptors were strongly induced in wild type and *Tpl2*^-/-^ keratinocytes treated with the phospho-EGFR inhibitor Gefitinib. HER3 has been implicated as a possible escape route in cancers resistant to traditional therapies [44,45]. Sustained HER3 signaling is a mode of resistance to EGFR-based therapies, and in certain cancers this HER3-dependent signaling can be driven by amplification of MET [46–48]. Additionally, upregulation of HER3 contributes to resistance to MAPK inhibitors in melanoma and thyroid carcinomas [49,50]. Although HER2 has no known ligand, we did find that treatment of wild type and *Tpl2*^-/-^ keratinocytes with HGF, the ligand for MET, could induce total HER2 and HER3 protein, although we didn’t test the mechanism by which this occurs. Others have shown that HGF can stimulate release of the EGFR ligand amphiregulin in squamous cell carcinoma, a process dependent on the MAPK protein Erk2 [51]. Thus, HGF may activate more than just the MET pathway, providing another avenue for crosstalk.

As described above, EGFR and MET are targetable RTKs, but when applied as single agents each of them regularly demonstrates only modest activity due to resistance and activation of compensatory signaling [48]. EGFR inhibitors used in clinical trials for the treatment of advanced cSCC only have modest clinical benefit, with 25–45% of the patients achieving partial responses and 0–18% obtaining complete remission [52]. Some have found that mutations in RAS can confer resistance to EGFR therapies [53]. Resistance to EGFR inhibitors can be caused by activation of RTK-mediated bypass pathways, including MET and HER-3, which can substitute for EGFR to maintain signaling output [54].

Since EGFR and MET activate many of the same downstream pathways, and can substitute for one another, combinatorial inhibition of EGFR/MET signaling has been suggested as a more effective treatment. Here, we tested whether dual EGFR/MET inhibition was more effective in reducing keratinocyte proliferation and indices of progression in wild type and *Tpl2*^*-/-*^cells. Both Gefitinib and Capmatinib decreased keratinocyte proliferation in wild type mice and Gefitinib reduced proliferation in *Tpl2*^*-/-*^cells. In regard to proliferation, combination treatment with Gefitinib + Capmatinib was not statistically different from Gefitinib alone. Further, combination treatment using Gefitinib + Capmatinib increased both endothelial tube formation and MMP-9 activity. In agreement with our data, other studies have also reported a lack of response in patients using EGFR/MET combination therapy [55–59]. In both phase I and phase II lung cancer studies, patients treated with a combination of EGFR and MET inhibitors had substantial toxicity and no improvement in overall survival compared to single agents [55–58]. Mechanistically, activation of additional parallel pathways, which can maintain signal output when EGFR and MET are inhibited, may explain why this combination wasn’t effective in keratinocytes. In Ras-transformed keratinocytes, we found that concomitant treatment with both EGFR/MET inhibitors heightened expression of numerous other RTKs including HER2/3/4, insulin receptor and IGF-1R. IGF-1R is a dimerization partner for insulin receptor, EGFR, HER2 and HER 3, all of which were induced in keratinocytes upon inhibition of MET and/or EGFR. Similar to our findings, others have reported that Gefitinib-resistant cells can activate IGF1R and EGFR/HER3 to stimulate downstream signaling through the PI3K/AKT and IGF-1R pathways [60]. Heterodimerization of IGF-1R with HER3 can also induce resistance to the EGFR/HER2 inhibitor Lapatinib, and heterotrimerization of IGF-1R with HER 2 and HER 3 provides resistance to trastuzumab [61,62]. With the ability of EGFR, MET, HER2/3, and IGF-1R to heterodimerize/ heterotrimerize with multiple different partners and compensate for one another, a large repertoire of possibilities exists to maintain survival signals and contribute to cSCC.

**Fig 8:**
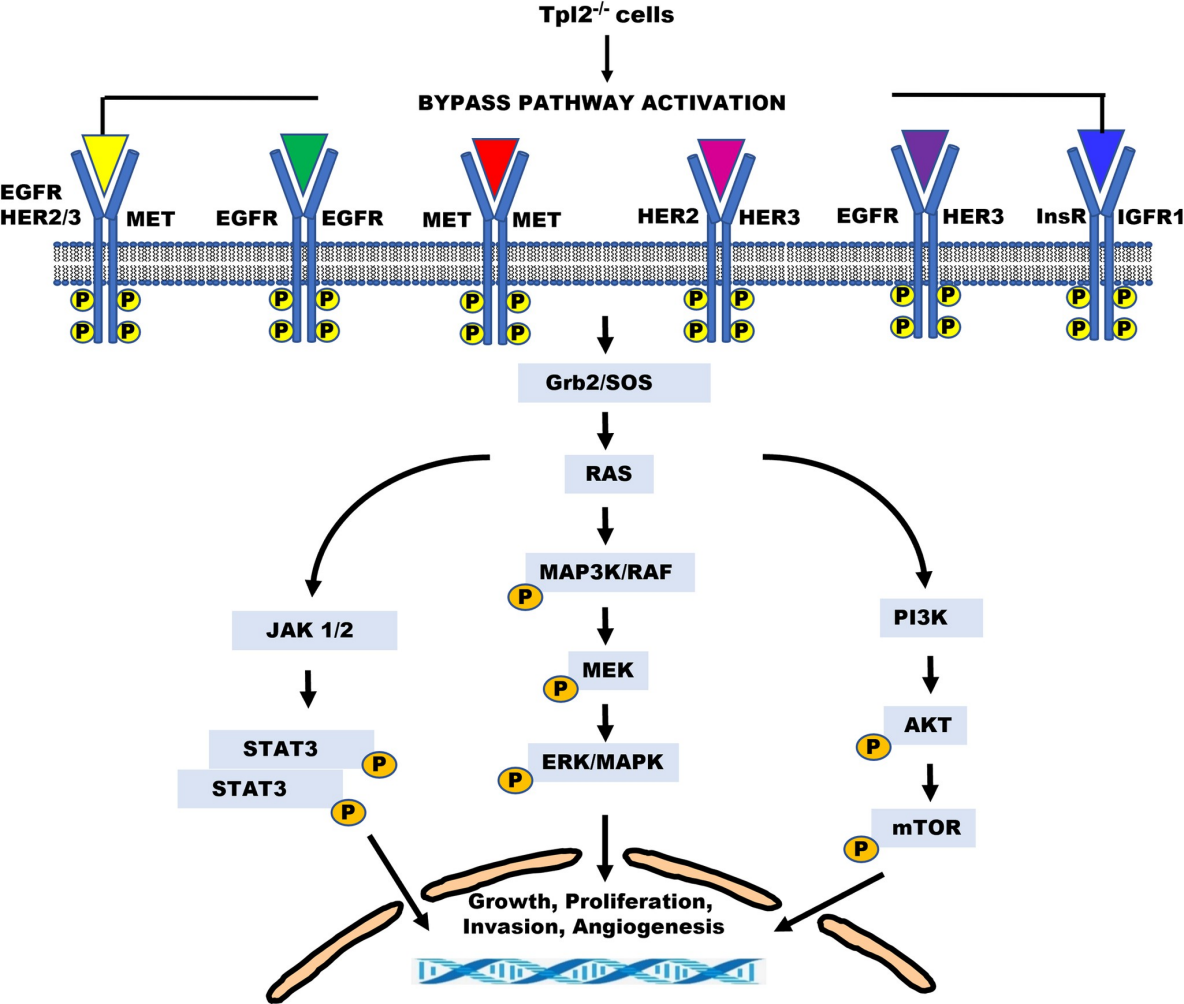
Proposed model of RTK bypass pathway activation in *Tpl2* ^-/-^ keratinocytes. Numerous RTKs become overactive in the absence of *Tpl2*. These RTKs can form multiple different partnerships with other RTKs and can compensate for one another, leading to sustained survival and progression signals.

In summary, we provide new insight into alternate signaling factors used by keratinocytes to bypass RTK inhibition and maintain growth and survival signaling. We find that *Tpl2* is part of an interconnected and convoluted web, involving RAS and multiple other RTKs (Fig 8). Having a better understanding of the redundancy and crosstalk in signaling cascades used by keratinocytes will provide new insight into potential resistance mechanisms that can contribute to cSCC.

## Materials and methods

### Wild type and transgenic mice

Male and female wild type (*Tpl2*^+/+^) and knockout (*Tpl2*^-/-^) C57Bl/6 mice were engineered as previously described [63]. The numbers of mice required for this experiment were the minimum numbers needed to achieve statistical power and protocols were performed in a manner to minimize animal pain and stress. *Tpl2*^-/-^ and *Tpl2*^+/+^ status was regularly confirmed by PCR. Mice were fed ad libitum an AIN-93M diet (*Tpl2* ^−/−^; *n*  =  8 or WT; *n*  =  12) or AIN-93M diet containing 44 mg/kg Capmatinib (*n*  =  8/genotype), an inhibitor of MET kinase activity. All groups were matched for age, weight and sex, monitored daily for general appearance, and weighed weekly. WT and *Tpl2*^*-/-*^ mice were initiated with 7,12-dimethylbenz(a)anthracene (DMBA; 100 μg/200 μl acetone) and promoted for 20 weeks with twice weekly application of TPA painted on the skin (10 μg/200 μl acetone). Tumor-bearing animals were individually housed to avoid injury to the tumor sites. Animals were euthanized 52 weeks after the date of initiation, or at an earlier time point if tumor size reached 20mm in any one dimension, the tumor became infected, the tumor affected the animal’s ability to eat, urinate or defecate, impaired ambulation, or the animal was deemed moribund by the veterinary staff. Mice were euthanized by exposure to carbon dioxide, using decapitation as a confirmatory secondary euthanization method. All tumors used in this study underwent histological examination in a blinded fashion by a certified pathologist (Mass Histology Services, Worcester, MA) to determine tumor type.

### Primary keratinocyte isolation and treatment

Primary keratinocytes were isolated from *Tpl2*^-/-^ and wild type mice pups at 1–4 days of age according to standard protocols [64]. Keratinocytes were grown in PromoCell keratinocyte growth media (VWR, Philadelphia, PA) containing hormone supplements, Penicillin-Streptomycin (10,000U/ml) and low (0.05mM) calcium. For some experiments keratinocytes were infected with v-*ras*^Ha^ retrovirus as described elsewhere and/or treated with the p-MET inhibitor Capmatanib (INCB28060; Selleck Chemicals, Houston, TX), HGF (R&D Systems, Minneapolis, MN), the phospho-EGFR inhibitor Gefitinib (Selleck Chemicals), or drugs in combination [64]. For *in vitro* drug combination studies, keratinocytes were treated with 2nM Capmatinib +/- 1uM Gefitinib for 2 hours followed by application of 20ng/mL HGF or vehicle (HGF; R&D Systems, Minneapolis, MN) for 15 minutes (for phosphorylation studies) or 24 hours (total protein) prior to protein isolation. For conditioned media experiments, media was collected from treated keratinocytes 18 hours post-treatment. After centrifugation, amount of total supernatant used was normalized to keratinocyte number.

### Proliferation experiments

2.5 x 10^4^ v-*ras*^Ha^-transduced wild type or *Tpl2*^-/-^ keratinocytes were plated in quadruplicate in opaque 96-well plates. Cells were grown in normal supplemented keratinocyte media or supplemented media containing 1uM Gefitinib, 2nM Capmatinib, or both Gefitinib and Capmatinib for 24 hours. Viability was measured using CellTiter-Glo Luminescent Cell Viability Assay (Promega, Madison, WI, USA) per manufacturer’s instructions.

### Western blotting

Total protein lysates were prepared from pooled untreated or treated keratinocytes using RIPA buffer containing Halt protease/phosphatase inhibitors in accordance with the manufacturer’s protocol (Thermo Fisher Scientific, Rockford, IL, USA). 25 μg of protein was electrophoresed using 4–12% gradient SDS-polyacrylamide gels, transferred to PVDF membrane and blocked with 5% BSA. Primary antibodies for pEGFR/EGFR, MET, pHER2/HER2, pHER3/HER3, pAKT/AKT, STAT3, pmTOR/mTOR, B-Actin were purchased from Cell Signaling and used at 1:1000. p-MET was purchased from Abcam and used at 1:1000. Anti-rabbit HRP secondary antibodies (Cell Signaling Technology), followed by West Dura Chemiluminescence substrate (Thermo, Rockland, IL) were used for signal detection. Bands were quantified using NIH Image J and normalized to the densitometry for the respective housekeeping gene. Western blots used pooled keratinocyte protein (n = 4–6 mice/pool) and were repeated a minimum of three times.

### Phospho-Receptor Tyrosine Kinase (RTK) assay

The Phospho-RTK assay was done in accordance to R&D Biosystem RTK Assay Kit Protocol. The kits contained 4 array membranes that had been pre-spotted with 39 different phosphorylated RTK antibodies, along with positive and negative controls. For this assay, 8 different samples were used: wild type *v-ras*^*Ha*^-infected, wild type *v-ras*^*Ha*^-infected + Capmatinib, wild type *v-ras*^*Ha*^*-*infected + Gefitinib, wild type *v-ras*^*Ha*^-infected + Capmatinib and Gefitinib, as well as the corresponding *Tpl2*^-/-^ samples. After incubating membranes with cell lysates containing 200ug of protein, anti-Phospho-Tyrosine-HRP Detection Antibodies were added for 2 hours at room temperature and then a Chemi Reagent Mix was used for detection. The membranes were imaged using ChemiDoc-it UVP imaging system. Densitometry was performed using NIH ImageJ and protein levels normalized to positive controls.

### Immunohistochemistry

Immunohistochemistry was performed as previously described [28]. 4μm sections were cut and stained with hematoxylin and eosin (H&E). Primary antibodies for p-EGFR (Abcam; Cambridge, MA) and anti-rabbit secondary antibodies (Cell Signaling; Danvers, MA) were used. Sections came from a minimum of three individual mice per treatment. Representative areas were photographed at 10 and 50x magnification.

### Zymography

Zymography was performed as previously reported [27]. Briefly, harvested cell-free conditioned media was electrophoresed on 10% tris-glycine gels containing 0.1% gelatin. Zymogram gels were incubated in zymogram renaturing buffer, developing buffer, stained with Coomassie blue G-250, and de-stained in deionized water. Bands were quantified using NIH Image J.

### Tube formation assay

Tube formation assays, an *in vitro* measure of angiogenesis, were conducted as previously described [39]. Briefly, 3B-11 endothelial cells were serum-starved in DMEM with 0.2% FBS overnight, Calcein-stained (Life Technologies; Grand Island, NY), plated onto Basement Membrane Extract (BME), and exposed to pooled conditioned media from treated or untreated normal or *v-ras*^*Ha*^-transduced keratinocytes. The tube network grew for 6–9 hours before paraformaldehyde fixing and then imaged using a fluorescence microscope (Olympus; Center Valley, PA). The tube network (number of nodes/meshes/segments) was quantified using ImageJ Angiogenesis Analyzer Plugin.

### Statistical analyses

Data was tested for normality, model assumptions were checked, and the data were analyzed with SPSS software. Tube formation assays, zymography and proliferation studies examining genotype and drug effects were analyzed through two-way ANOVA with Tukey’s post-hoc test. Significance for all analyses was assumed at a *p*-value of 0.05 or less. Significance values of *p* ≤ 0.05 are indicated in figures with a single asterisk (*), *p* ≤ 0.01 with a double asterisk (**), and *p* ≤ 0.001 with a triple asterisk (***).

## Ethics statement

All mice were bred and maintained at The American University animal facility (Washington, D.C., USA) in compliance with the National Institutes of Health and ARRIVE guidelines and an approved Institutional Animal Care and Use Committee (IACUC) protocol.

## Supporting information

S1 Fig Raw images for Western blots. https://mfr.osf.io/render?url=https%3A%2F%2Fosf.io%2Ff7wxg%2Fdownload.
(PDF)

S2 Fig Western blot densitometry. Densitometry of Western Images from Fig 1A (A), Fig 1B (B), and Fig 2 (C). https://mfr.osf.io/render?url=https%3A%2F%2Fosf.io%2Fam2tb%2Fdownload.
(TIF)

S3 Fig Tube formation assay. Endothelial tube formation assay. 120,000 3B-11 mouse endothelial cells were serum starved and plated with conditioned media from wild type or *Tpl2*-/- keratinocytes that had been treated with 20ng/ml HGF, HGF+ 2nM Capmatinib, 1uM Gefitinib, or HGF/Cap/Gef. This is a repeat of Fig 5, with higher numbers of endothelial cells in order to develop a more extensive network. The number of segments, nodes and mesh were calculated using Image J with Angiogenesis Plugin and statistics determined using a two-way ANOVA. https://mfr.osf.io/render?url=https%3A%2F%2Fosf.io%2Fsnuew%2Fdownload.
(TIF)

S1 Dataset Minimal data set for figures and graphs used in manuscript. https://mfr.osf.io/render?url=https%3A%2F%2Fosf.io%2Fmpw7v%2Fdownload.
(CSV)
