# Supplementary material for: Loss of Tpl2 activates compensatory signaling and resistance to EGFR/MET dual inhibition in v-RAS transduced keratinocytes
Source: PLoS One. 2022 Mar 24;17(3):e0266017. doi: 10.1371/journal.pone.0266017 (PMC8947257; doi:10.1371/journal.pone.0266017)
Supplement: S1 Fig — https://mfr.osf.io/render?url=https%3A%2F%2Fosf.io%2Ff7wxg%2Fdownload. (PDF) [file pone.0266017.s001.pdf]

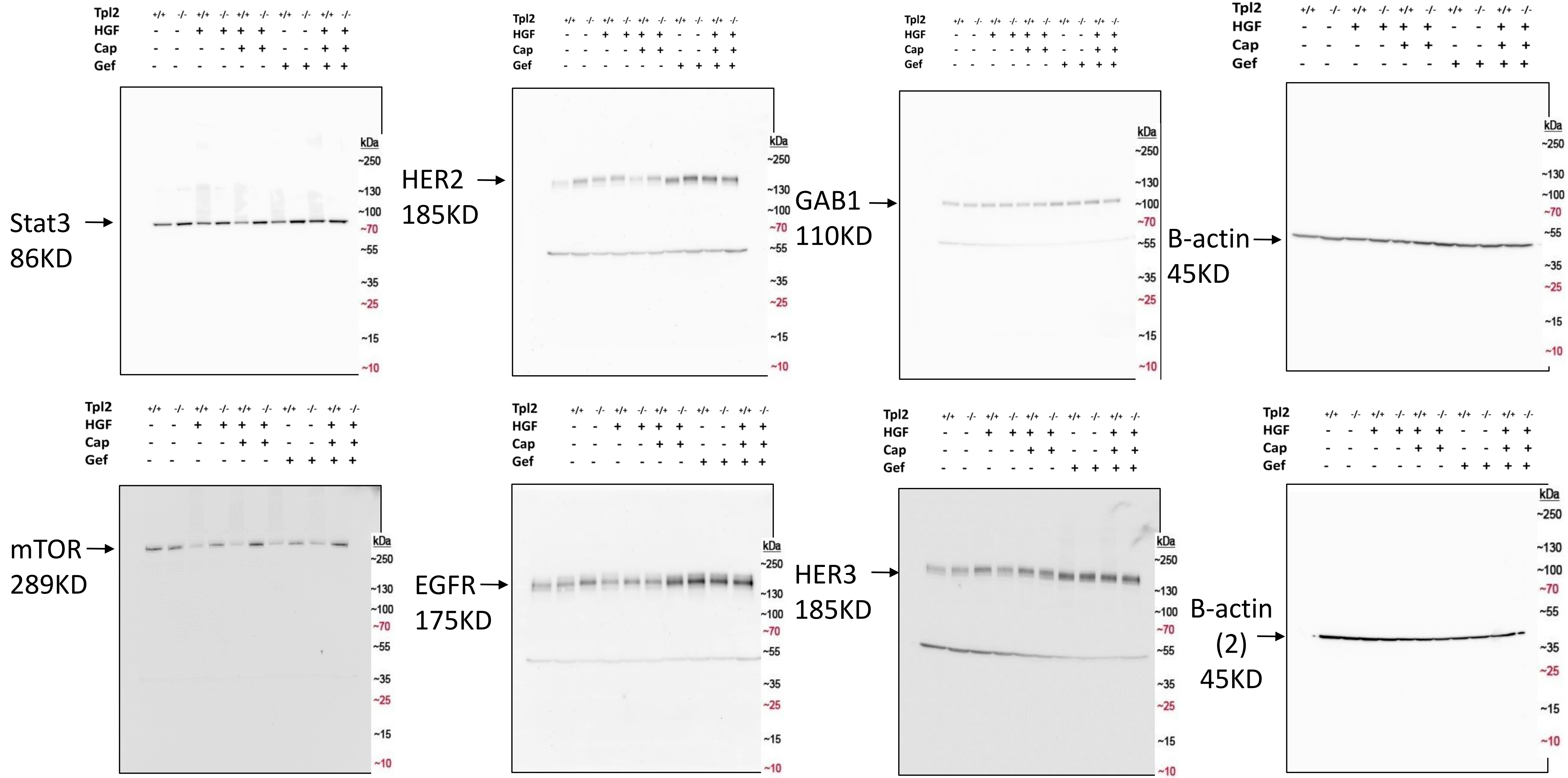

S1 Raw images. Full-length gels from Western blot cropped images displayed in Figure 1A. All blots were imaged with a UVP ChemiDoc-It imager with 4x4 binning.

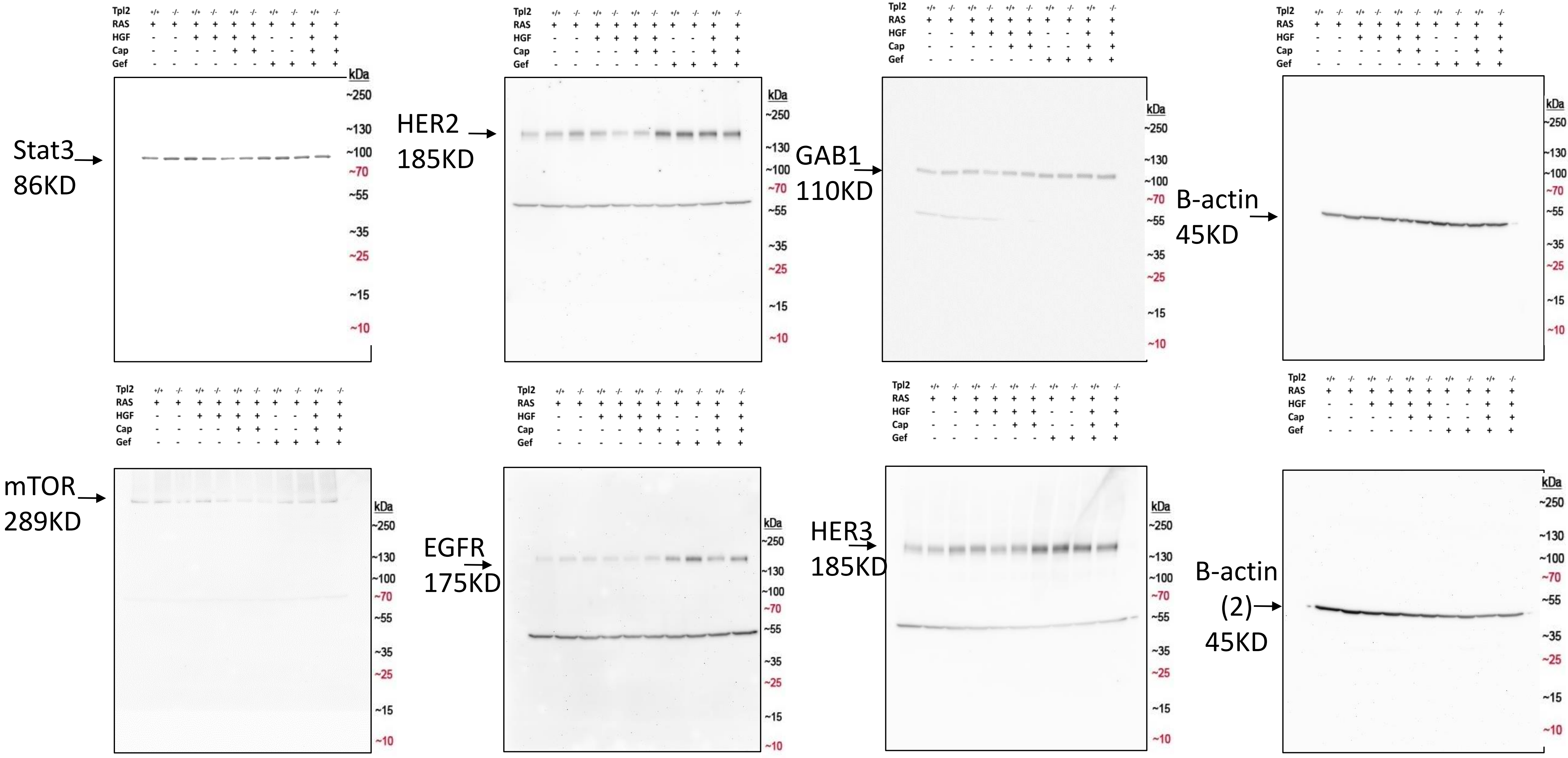

S1 Raw images. Full-length gels from Western blot cropped images displayed in Figure 1B. All blots were imaged with a UVP ChemiDoc-It imager with 4x4 binning.

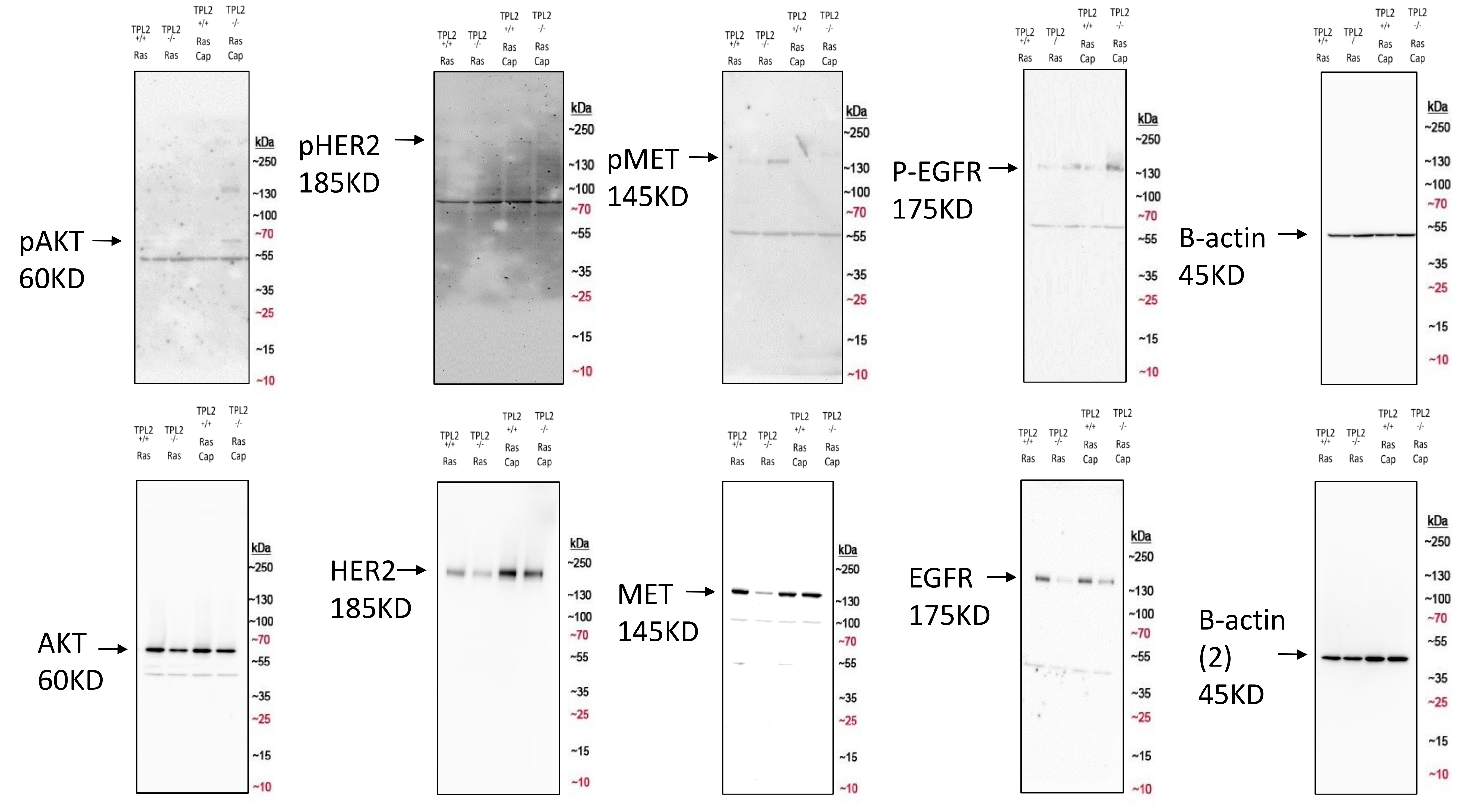

S1 Raw images. Full-length gels from Western blot cropped images displayed in Figure 2. All blots were imaged with a UVP ChemiDoc-It imager with 4x4 binning.
