# Supplementary figures and images for: Loss of Tpl2 activates compensatory signaling and resistance to EGFR/MET dual inhibition in v-RAS transduced keratinocytes
Source: PLoS One. 2022 Mar 24;17(3):e0266017. doi: 10.1371/journal.pone.0266017 (PMC8947257; doi:10.1371/journal.pone.0266017)

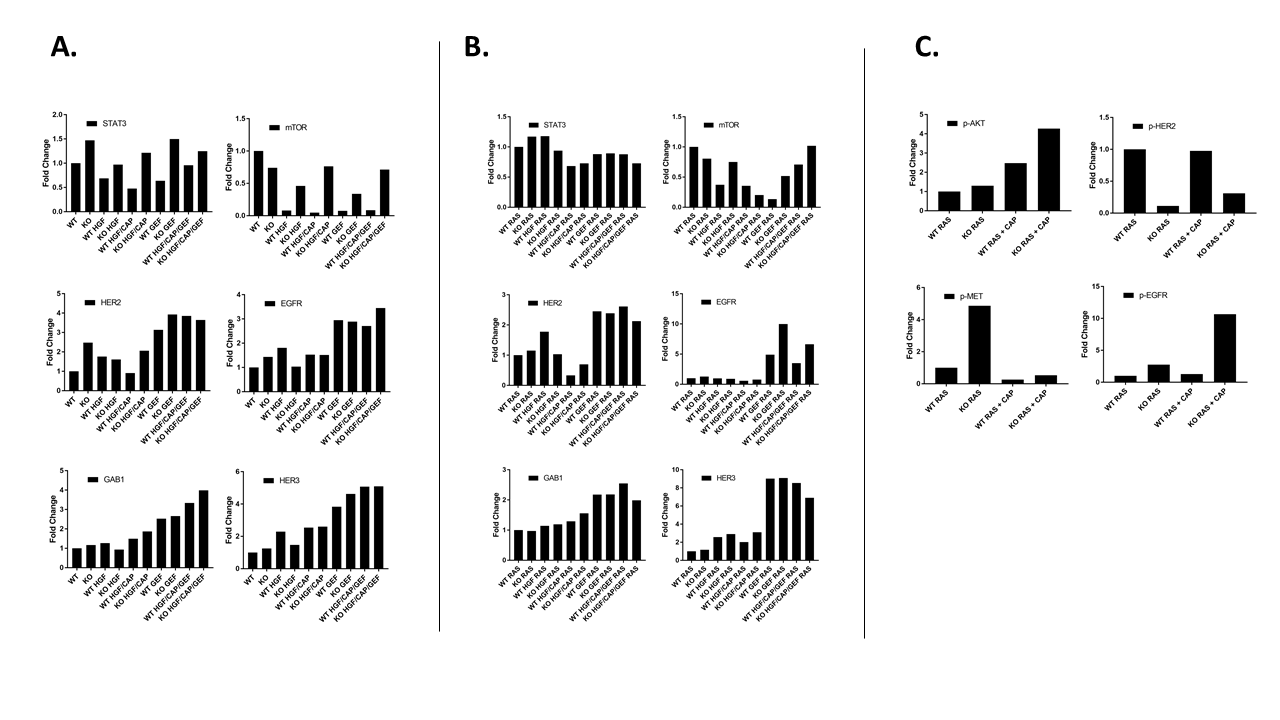

Supplement: S2 Fig — Densitometry of Western Images from Fig 1A (A), Fig 1B (B), and Fig 2 (C). https://mfr.osf.io/render?url=https%3A%2F%2Fosf.io%2Fam2tb%2Fdownload. (TIF) [file pone.0266017.s002.tif]

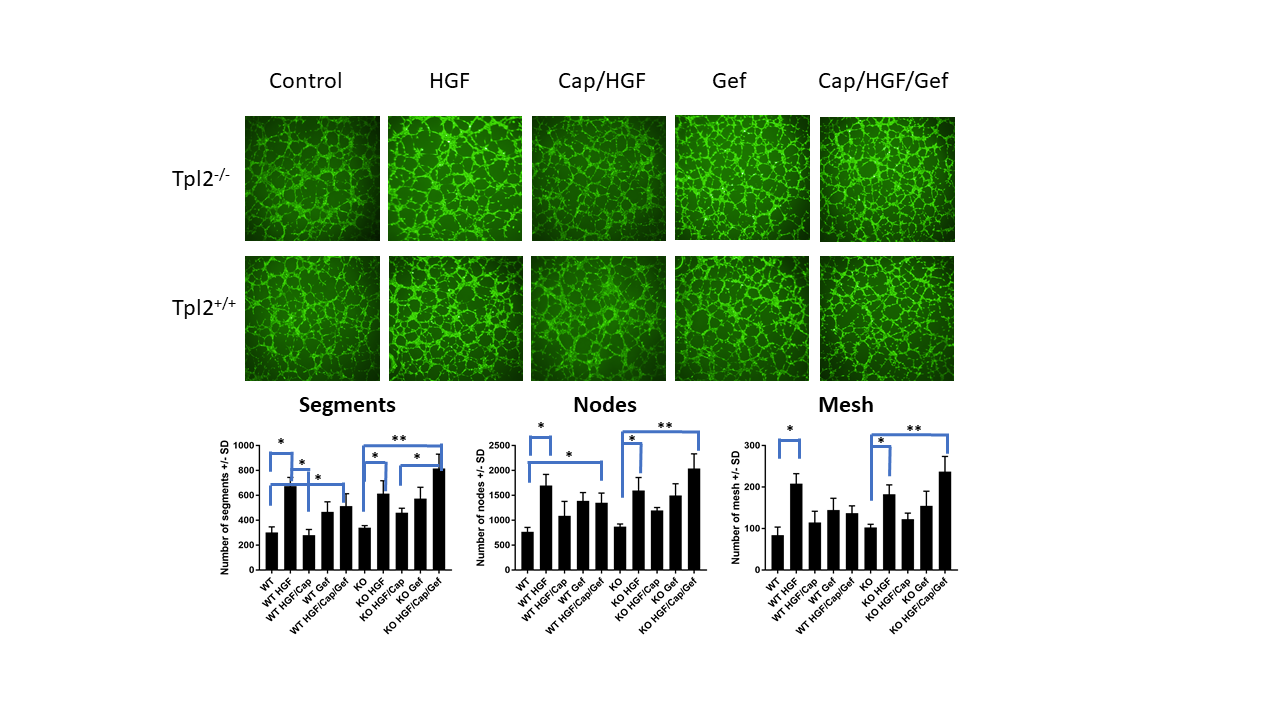

Supplement: S3 Fig — Endothelial tube formation assay. 120,000 3B-11 mouse endothelial cells were serum starved and plated with conditioned media from wild type or Tpl2-/- keratinocytes that had been treated with 20ng/ml HGF, HGF+ 2nM Capmatinib, 1uM Gefitinib, or HGF/Cap/Gef. This is a repeat of Fig 5, with higher numbers of endothelial cells in order to develop a more extensive network. The number of segments, nodes and mesh were calculated using Image J with Angiogenesis Plugin and statistics determined using a two-way ANOVA. https://mfr.osf.io/render?url=https%3A%2F%2Fosf.io%2Fsnuew%2Fdownload. (TIF) [file pone.0266017.s003.tif]
